# Comparative study of the efficacy of dexmedetomidine and fentanyl on anxiety and pain of parturients with different COMTva1158met genotypes

**DOI:** 10.1186/s12871-022-01628-2

**Published:** 2022-03-28

**Authors:** Li-Jia Tang, Jiang Han, Yi-Juan Feng, Cui-Xia Pu, Ying Zhang

**Affiliations:** Department of Anesthesiology, Hospital (T.C.M) Affiliated to Southwest Medical University, Chunhui Road 182#, Luzhou, Sichuan, 646000 China

**Keywords:** Catechol O-methyltransferase Gene Polymorphism, Dexmedetomidine, Fentanyl, Labor Anxiety, Labor Analgesia

## Abstract

**Objective:**

This study investigates the effects of *COMTval158met* gene polymorphism on maternal anxiety and pain during delivery and on the analgesic and anxiety efficacy of dexmedetomidine during delivery.

**Methods:**

Sixty-one pregnant women, who were hospitalized in our hospital from January to November of 2016 were recruited and randomly divided into two groups, F and D groups. The pregnant women in the F group were given labor analgesia with ropivacaine combined with fentanyl. The pregnant women in the D group were given labor analgesia with ropivacaine combined with dexmedetomidine. Before and after labor analgesia, the genotype of *COMT* in the blood from two groups was detected, and the situation of labor anxiety and analgesia was analyzed. Then, the relationship between labor anxiety, analgesia, and *COMT* polymorphism was analyzed.

**Results:**

In the 61 pregnant women, there were 30 women of wild homozygotes (GG) of *COMT*, 22 women of mutant heterozygotes (GA), and nine women of mutant homozygotes (AA), the mutation rate of allele A was 23.77%. The anxiety status score, anxiety trait score, and pain score in the AA genotype were significantly higher than those in the GG and GA genotype (*p* < 0.05). There was a significant difference in the efficacy of GG and AA genotypes between groups D and F for treating labor anxiety (*p* < 0.05), the efficacy of group D was better than that of group F in treating delivery anxiety, there was no significant difference in anxiety scores between the two groups in GA genotypes (*p* > 0.05); there was no significant difference in pain between group D and F in GG, GA, and AA genotypes (*p* > 0.05). There was no significant difference in pain and anxiety scores between the three genotypes in group D (*p* > 0.05), there was significant difference in pain scores among the three genotypes in group F (*p* < 0.05), but there was no significant difference in anxiety (*p* > 0.05).

**Conclusions:**

The mutation of the *COMTval158met* gene leads to increased anxiety and pain during childbirth. The effect of dexmedetomidine on the anxiety of GG and AA genotypes is better than that of fentanyl, and the mutation of the *COMTval158met* gene has no impact on dexmedetomidine effect.

## Introduction

Anxiety and pain during childbirth are common phenomena. They can change hormone secretion (for example, adrenocorticotropic hormone, cortisol, catecholamines, and endorphin) and a reduction in pain threshold in pregnant women; additionally, aggravation of pain affects emotions [1]. The above phenomena result in a fear-tension-pain syndrome and form a vicious circle for pregnant women. Eventually, these adverse factors cause complications during childbirth, such as increased dystocia and cesarean section rates [2]. So far, it is generally believed that epidural analgesia is an effective way of relieving labor pain [3]. With the accomplishment of the human genome project, research from the angle of the gene to consider anxiety and pain treatment is drawing more and more attention. 

The difference in nucleotide sequence between individuals in a population is called gene polymorphism. Human genetic polymorphism plays a vital role in explaining the differences in human body diseases, drug tolerance and susceptibility, the diversity of clinical manifestations of the disease, and the body's response to drug therapy [4]. Previous studies have shown that *COMT* polymorphism could regulate psychological and stress factors by changing the activity of catechol oxymethyltransferase (*COMT*), thus, affecting the human perception of pain [5]. Therefore, *COMT* polymorphism is one a genetic factor that determines different endurance and response patterns to pain or other stress [6].

Formerly, Opioid, a clinically commonly used intraspinal adjuvant, has no anti-anxiety effect and with individual differences in efficacy[7]. As a highly selective alpha 2 receptor agonist, dexmedetomidine can act on alpha 2α adrenergic receptor in the locus coeruleus, inhibit sympathetic nerve activity, and has obvious sedative and analgesic effect, similar to natural sleep and without respiratory inhibition [8]. Additionally, dexmedetomidine can also inhibit sympathetic nerve excitation to achieve the purpose of anti-anxiety [9]. Dexmedetomidine can also be coordinated with local anesthetics to prolong pain time and decrease anesthetic dosage without damaging nerves [10]. Whether *COMT* polymorphism affects pregnant women's labor anxiety and analgesia has not been reported. This study explored the effect of *COMT* polymorphism on fentanyl and dexmedetomidine in labor anxiety and analgesia.

## Materials and methods

### Patients

Sixty-one pregnant women were recruited for this study, they were admitted and hospitalized in our hospital from January to November of 2016. Before delivery analgesia, the *COMT* genotyping had been tested. The age of all pregnant women ranged from 20 to 32 years old and the gestational week was between 38 and 41 weeks. American Society of Anesthesiologists physical status I and II were selected. Single birth primipara, no head and pelvis disproportionate (CPD), prenatal care without pathological obstetrics, no severe medical diseases, no drinking history, smoking, psychosis, contraindication of epidural anesthesia, etc. This study excluded pregnant women who gave birth through the uterine incision for several reasons.

### COMT gene detection

**Fig. 1:**
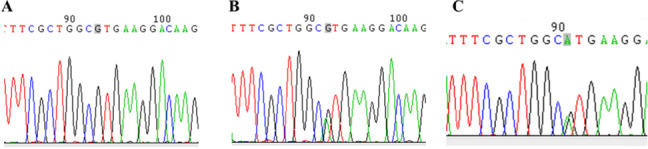
The polymorphism of the *COMTval158met* gene was identified using polymorphic site direct PCR sequencing. The orange-labeled trinucleotide is the codons corresponding to amino acid 158 (Val), and the sequencing peak corresponding to the sequence is sequenced. The gray-marked base is the polymorphic site

During the first month before labor analgesia, 5-ml peripheral blood was collected, which was placed in the anticoagulant tubes, and stored at − 80℃. And then, the whole blood DNA was extracted using DNA Extraction Kit. PCR and restriction fragment length polymorphism (RFLP) were used to detect the expression of *COMT* gene. Briefly, with the help of upriver primer 5'-ACTGTG-GCTACTCAGCTGTG-3 and downstream primer-5'-CCTTTTTC-CAGGTCTGACAA-3' (synthesized by Shenggong biology and technology co. ltd). The reaction system consisted of such reagents as 10 × buffer (3-μL), dNTP mixture (0.5-μL), DNA template (2-μL), upriver and downstream primer( each for 0. 5-μL), Taq DNA polymerase, with the addition of distilled water until the total volume reaching 30-μL. The PCR reaction condition was set up as follows:: pre-denaturation at 94℃ for five minutes, denaturation at 94℃ for 30 s, reannealing at 55℃ for 45 s, and elongation at 72℃ for 60 s, 35 cycles, the products were stored at 4℃. The endonuclease NIa III was used for enzyme digestion of PCR products. A total of 5-μL products of enzyme produce were used for staining using GeneFinder nucleic acid dye. The gel electrophoresis of 1% agarose gel under 100 v was conducted for 30 min; with the help of molecular standard 50-bp DNA Ladder, the PCR amplified products underwent sequencing and genotyping (Fig. 1). According to the genotype, the wild-type homozygote (Val/Val) was defined as GG, the mutant homozygote (Met/Met) was defined as an AA, and the mutant heterozygote (Val/Met) was defined as GA.

According to the strategy for labor analgesia, the maternity was randomly divided into two groups: dexmedetomidine and fentanyl groups.

## Strategy for labor analgesia

After regular uterine contraction, the pregnant women were given an intravenous infusion of 500–1000-ml 0.9% normal saline. When the cervical dilatation was enlarged by 3–4-cm, an epidural puncture in the intervertebral space L2–L3 was performed by an anesthesiologist. After a successful puncture, the pregnant women in the fentanyl group, the intervertebral space, were injected with 0.08% ropivacaine 6-ml + 6-ug fentanyl. Then, labor analgesia was administered through the same route: 80-mg 0.08% ropivacaine + 100-g fentanyl + normal saline to 100-ml and the background dose was 8-ml/h. After a successful puncture, the pregnant women in the fentanyl group, the intervertebral space, were injected with 0.08% rocapabine 6-ml + 6-ug dexmedetomidine, and then, the labor analgesia was administered through the same route and doses of medicine mentioned before.

## Anxiety evaluation of maternity

The anxiety scale was used to assess pregnant women receiving analgesia before and after delivery. Simultaneously, the filling requirements were explained in detail, allowing pregnant women to fill out a form within the painful interval. Score sheets were retrieved on site. According to the requirements of the rating scale, the standard score was obtained thorough conversion. The higher the score, the more serious the maternal anxiety patients showed. The normal value was < 50 scores, 50–59 scores were mild anxiety, 60–69 scores were moderate anxiety and > 70 scores was severe anxiety.

## Analgesia score

Before and after the labor analgesia, Visual Analogue Scale (VAS) was used for analgesia score. As for VAS Score, 0 score represents no pain, < 3 scores represents mild pain, which maternity could endure. When 4–6 scores represent maternity pain that influenced sleeping, could endure, while 7–10 scores represent severe pain that could not endure.

## Statistical analysis

The purpose of this study was to compare the differences in efficacy between the two groups, and score according to the main outcome indicators. Using PASS15.0 software, the sample size was calculated according to the sample size compared with the mean of the two samples. According to the previous observation results, the mean and standard deviation of anxiety scores of the two groups before and after intervention in GG genotype were 26 ± 3 in dexmedetomidine group and 22 ± 4 in fentanyl group. The class I error α of hypothesis test was 0.05. Using bilateral test, II error β was 0.2. the ratio of sample size between the two groups was 1:1. The sample size of each group was 14 people, that is, the total sample size was 28 people. Previous investigation found that the proportion of GG genotype in the population was about 50%, then the total population sample size was 56. In order to improve the testing efficiency, our final sample size was 61, exceeding the minimum sample size.

SPSS 13.0 software was used for data analysis. For normally distributed measurement data, they were expressed as $\overline{\mathrm{X}}\pm \mathrm{S }$. For comparing two sample means, a t-test was used. As for non-normal data, they were expressed as *M (Q*_*25*_ ~ *Q*_*75*_*).* When comparison was conducted between the two groups, the rank-sum test was employed for the non-normal distributed data. When comparison was conducted before, and after the treatment, t-test and rank-sum test was used.

## Results

### General status

In the 61 pregnant women, there were 30 women with wild homozygotes (GG) of *COMT*, 22 women with mutant heterozygotes (GA), and nine women of mutant homozygotes (AA), the mutation rate of allele A was 23.77%. According to drug grouping, there was no significant difference in culture degree and age in the fentanyl group and dexmedetomidine group (*p* < 0.05). According to gene grouping, there was no significant difference for culture degree and age among GG, GA and AA groups (*p* < 0.05), as indicated in Table 1.

**Table 1:** Comparison for the general status of patients in different groups

Grouping	Culture degree		$\chi^{{2}}$	*P*	Age ($\overline{{\text{X}}} \pm {\text{S}}$)	t/F	*P*
	College and below n(%)	College and above n(%)					
Drugs							
F group	18(58.06)	13(41.94)	1.575	0.21	24.68 ± 3.59	0.552	0.941
D group	22(73.33)	8(26.67)			25.20 ± 3.80		
Genotypes							
GG	20(66.70)	10(33.30)	6.00	0.05	23.87 ± 2.96	2.63	0.081
GA	12(54.5)	10(45.5)			25.95 ± 4.11		
AA	9(100)	0(0.0)			26.00 ± 4.93		

### Comparison of anxiety state and pain score in different genotypes before treatment

The anxiety state score, anxiety trait score, and pain score in AA genotype maternity was markedly higher than that of GG and AA, respectively (*p* < 0.05), as indicated in Table 2.

**Table 2:** Comparison of anxiety state and pain scores among different genotypes before treatment

Grouping	n	Anxiety state score(M, Q_25_ ~ Q_75_)	anxiety trait score(M, Q_25_ ~ Q_75_)	VAS score ($\overline{\mathrm{X}}\pm \mathrm{S }$)
GG	30	65.00(60.00 ~ 68.00)^*^	65.00(62.00–65.00)^*^	7.30 ± 1.06^*^
GA	22	68.00(61.50 ~ 68.25)^*^	66.50(61.50 ~ 68.00)^*^	7.64 ± 1.77^*^
AA	9	70.00(68.50 ~ 72.50)	70.00(67.00 ~ 71.00)	9.00 ± 1.00
*F/*$\chi^{{2}}$		15.98	15.14	8.38
*P*		< 0.001	0.001	0.001

^*^represents compared with AA, *p* < 0.05. *VAS* Visual Analogue Scale

#### Comparison of the efficacy of dexmedetomidine and fentanyl in different COMT genotype

##### Comparison of the efficacy of dexmedetomidine and fentanyl in GG genotype

In GG genotype parturients, there was a significant difference in anxiety state between dexmedetomidine and fentanyl (*p* < 0.003) and dexmedetomidine was better than fentanyl in an anxiety state. Still, there was no significant difference in pain (*p* > 0.05). as indicated in Table 3.

**Table 3:** Comparison of anxiety state and pain differences between dexmedetomidine and fentanyl in GG genotype patients

Grouping	n	Difference in anxiety state ($\overline{\mathrm{X}}\pm \mathrm{S }$)	Difference in VAS score(M, Q25 ~ Q75)
F group	15	26.40 ± 3.312	5.00(4.00 ~ 5.00)
D group	15	22.53 ± 4.486	5.00(4.00 ~ 6.00)
* t/z*		2.686	0.413
* P*		0.012	0.680

*VAS* Visual Analogue Scale

##### Comparison of the efficacy of dexmedetomidine and fentanyl in GA genotype

There was no significant difference in anxiety and pain between dexmedetomidine and fentanyl in parturients with GA genotype (*p* > 0.05), as indicated in Table 4.

**Table 4:** Comparison of anxiety state and pain differences between dexmedetomidine and fentanyl in patients with GA genotype

Grouping	n	Difference in anxiety state score ($\overline{\mathrm{X}}\pm \mathrm{S }$)	Difference in VAS score (M, Q_25_ ~ Q_75_)
F group	11	23.09 ± 7.661	5.00(4.00 ~ 6.00)
D group	11	25.00 ± 5.060	6.00(5.00 ~ 6.00)
t/z		0.690	1.010
* P*		0.498	0.313

##### Comparison of the efficacy of dexmedetomidine and fentanyl in AA genotype

There was a statistically significant difference in the therapeutic effect of dexmedetomidine and fentanyl in the anxiety state among AA genotype parturients (*p* < 0.05), and the therapeutic effect of dexmedetomidine was better than that of fentanyl, but there was no statistically significant difference in pain (*p* > 0.05), as indicated in Table 5.

**Table 5:** Comparison of anxiety state and pain differences between dexmedetomidine and fentanyl in AA genotype patients

Grouping	n	Difference in anxiety state ($\overline{\mathrm{X}}\pm \mathrm{S }$)	Difference in VAS score (M, Q_25_ ~ Q_75_)
F group	4	25.00 ± 3.202	6.00(4.50 ~ 6.75)
D group	5	19.40 ± 3.847	6.00(5.50 ~ 6.50)
t/z		2.641	0.135
*P*		0.033	0.893

#### The effect of different genotypes on the efficacy of dexmedetomidine and fentanyl.

##### The effect of different genotypes on the efficacy of dexmedetomidine

There was no statistical significance in the treatment effect of dexmedetomidine on anxiety and pain among the three genotypes of parturients (*p* > 0.05), as indicated in Table 6.

**Table 6:** Comparison of anxiety and pain difference between patients with different genotypes before and after dexmedetomidine treatment

Grouping	n	Difference in anxiety state ($\overline{{\text{X}}} \pm {\text{S}}$)	Difference in VAS score ($\overline{{\text{X}}} \pm {\text{S}}$)
GG	15	26.40 ± 3.312	5.00 ± 0.845
GA	11	22.81 ± 4.069	6.00 ± 1.044
AA	4	25.75 ± 3.20	5.75 ± 1.258
F		1.250	0.951
*P*		0.302	0.399

##### Effects of different genotypes on the efficacy of fentanyl

There was a statistically significant difference in the treatment effect of fentanyl for the three genotypes of parturients in pain (*p* < 0.05), but there was no statistically significant difference in anxiety state (*p* > 0.05). as indicated in Table 7.

**Table 7:** Comparison of anxiety and pain differences between patients with different genotypes before and after fentanyl treatment

Grouping	n	Difference in anxiety state ($\overline{{\text{X}}} \pm {\text{S}}$)	Difference in VAS score ($\overline{{\text{X}}} \pm {\text{S}}$)
GG	15	22.53 ± 4.485	4.73 ± 1.163
GA	11	25.00 ± 5.060	5.54 ± 0.688
AA	5	19.40 ± 3.847	6.00 ± 1.258
F		2.622	3.916
*P*		0.090	0.032

## Discussion

### Comparison of the efficacy of dexmedetomidine and fentanyl in different COMT genotype

In the study of parturients with GG, GA, and AA genotypes, it was found that there was a significant difference in the efficacy of dexmedetomidine and fentanyl for treating anxiety between GG and AA genotypes (*p* < 0.05), respectively, and the efficacy of dexmedetomidine was better than that of fentanyl. In terms of pain treatment, there was no significant difference in the efficacy of dexmedetomidine and fentanyl for treating pain among parturients with GG, GA, and AA genotypes (*p* > 0.05). Therefore, it was believed that in the formulation of drug use for delivery, especially those with severe anxiety and preoperative genetic test of GG and AA, the use of dexmedetomidine can control labor anxiety more effectively without affecting the analgesic effect.

### Effects of different COMTval158met genotypes on the efficacy of dexmedetomidine and fentanyl

This study showed that *COMT*val158met gene polymorphism affected the degree of anxiety and pain in parturients before labor analgesia. After using dexmedetomidine, the efficacy of dexmedetomidine on three genotypes were compared. It was found that there was no significant difference in pain and anxiety between GG, GA, and AA genotypes, while *COMTval158met* gene polymorphism did not affect the efficacy of dexmedetomidine in labor analgesia. After using fentanyl for analgesia, it was found that there were differences in pain among the three genotypes. It showed that the degree of pain relief of parturients with the *COMTval158met* mutant AA gene was better than that of AG and GG genes. Fentanyl is a synthetic strong analgesic anesthetic and an opioid receptor agonist. Its analgesic activity is similar to morphine, and its intensity is 60–80 times higher than that of morphine [11]. Previous studies have also confirmed a correlation between *COMTval158met* gene polymorphism and postoperative opioid dosage. *COMTval158met* mutation reduces postoperative opioid dosage in patients [12]. With the continuous improvement of human group-based programs, some scholars believe that genetic testing is helpful to provide the accuracy of drug therapy.

This study used polymerase chain reaction-restriction fragment length polymorphism (PCR–RFLP) to detect the *COMTval158met* gene in 61 parturients. The results showed the presence of 30 cases of wild homozygote GG type, 22 cases of mutant heterozygote GA type, and 9 cases of mutant homozygous AA type. The mutation rate of allele A was 23.77%. The anxiety state score, anxiety trait score, and pain score of women with AA genotype were higher than those with GG and GA genotypes. Presently, it is believed that *COMTval158met* gene polymorphism can change the *COMT* activity [13]; *COMTval158met* gene mutation reduces *COMT* activity, leads to reduced catecholamine degradation in vivo, increases catecholamine content, and even leads to an abnormal anxiety state [14]. Simultaneously, *COMT* activity is an essential factor in regulating pain sensitivity, which participates in the central sensitization and pain relief process of catecholamines [15, 16]. Therefore, this study suggests that the degree of anxiety and pain during delivery is related to the polymorphism of the *COMTval158met* gene. The mutation of the *COMTval158met* gene changes the activity of *COMT*, which decreases catecholamine degradation and the increase in catecholamine content. Finally, it leads to a rise in anxiety and pain during delivery. Dexmedetomidine is a synthetic sedative and analgesic drug, which can inhibit the impulse of anterior horn sympathetic nerve cells and reduce sympathetic tension [17]. Simultaneously, dexmedetomidine activates vagus-cardiac reflex and baroreceptor reflex, inhibits the release of norepinephrine at the end of compassion, and reduces the concentration of catecholamine in blood [18]. In GG and AA genotypes. Dexmedetomidine has a better effect on labor anxiety under the same analgesic effect, and *COMTval158met* gene polymorphism does not affect the efficacy of dexmedetomidine in labor analgesia. Still, because of its high price, it is widely used clinically. Detection of *COMTval158met* gene before an operation, different drug regimens for different genotypes of parturients, dexmedetomidine for AA and GA parturients, can better improve labor anxiety under the same analgesia. In contrast, GA parturients can use fentanyl with relatively low price. Therefore, it is essential to study multi-mode, multi-center, large sample, and combined polygenes of analgesia.

Shortcomings of this study 1. Only the mutation of the *COMTval158met* gene was analyzed, without excluding the interference of other related genes, so the effect of genes on analgesic delivery efficacy could not be comprehensively evaluated.2. Because of the low mutation rate of genes, the proportion of AA samples in the same sample size was relatively low.

## Data Availability

The datasets used and/or analysed during the current study are available from the corresponding author on reasonable request.

## Abbreviations

HGP: Human Genome Project
COMT: Catechol Oxymethyltransferase
ASA: Anesthesiologist
VAS: Visual Analogue Scale
SAS: Used Anxiety Scale
